# Listener-level aesthetic appraisal in personalized music-listening interventions for older adults: a closed-loop framework

**DOI:** 10.3389/fpsyg.2026.1950140

**Published:** 2026-10-01

**Authors:** Ruosi Guo, Yu Wang, Fanyu Meng

**Affiliations:** College of Art, Hebei Agricultural University, Baoding, China

**Keywords:** aesthetic appraisal, closed-loop framework, listener feedback, musical emotion, music-listening intervention, older adults, personalized music

## Abstract

Music-listening interventions yield modest average benefits in later life, but substantial heterogeneity remains unexplained. Existing systems commonly organize music by symptom labels, mood, acoustic features, cultural categories, expert judgment, or preference, and often infer listener response from liking, skips, completion, duration, or mood check-ins. We define listener-level aesthetic appraisal as a person-specific appraisal profile accumulated across listening events and operationalized through event-specific evaluations, distinct from preference, familiarity, autobiographical salience, engagement, exposure, adherence, and therapeutic outcome. For repeated use in personalized music-listening interventions, we propose a provisional, low-burden three-item assessment of meaningfulness, emotional or aesthetic resonance, and overall satisfaction. Each item is rated from 0 (“not at all”) to 4 (“very much”) immediately after listening. Direct appraisal measurement is intended for listeners able to provide interpretable self-report, independently or with support; a separate observational care pathway is proposed when such self-report is unavailable, without treating observation as first-person appraisal. We then outline a two-component closed loop comprising a quality-controlled repertoire-side candidate space and a listener-side component with event-specific appraisal, illustrative hierarchical ordinal modelling, Bayesian updating, constrained probabilistic selection, and separate computational and participant-level stopping rules. The framework is illustrative and testable, not a fitted or validated algorithm. We propose prospective tests of incremental predictive value, adaptive versus static matching, and the balance among aesthetic fit, intervention goals, safety, autonomy, consent, and assessment burden.

## 1 Introduction

Music-listening interventions are used to support mood, sleep, stress regulation, and quality of life, and the aggregate evidence is encouraging but strikingly uneven. A Cochrane review of music-based therapeutic interventions in dementia found moderate-certainty evidence of reduced depressive symptoms (SMD −0.27) but little or no effect on agitation or aggression (van der Steen et al., 2018). Meta-analyses of music interventions for sleep in older adults report improvements on the Pittsburgh Sleep Quality Index of MD −2.64 (95% CI −3.76 to −1.53) and MD −3.37 (95% CI −4.24 to −2.50) in two independent syntheses (Wang et al., 2021; He et al., 2026). A network meta-analysis of music interventions for depression in older adults found that therapist-led active music therapy exceeding 60 min per week outperformed other configurations, indicating that nominally similar “music interventions” are not interchangeable (Dhippayom et al., 2022).

Two features of this evidence base motivate the present argument. First, the confidence intervals are wide and the pooled effects are modest relative to the between-study variability. Second, and more consequentially, averages of this magnitude are compatible with two very different realities: a uniformly small benefit for nearly everyone, or a mixture of strong responders and non-responders whose experiences cancel in the mean. The two possibilities carry opposite implications for practice—the first recommends broader delivery, the second recommends better matching—and distinguishing them requires knowing what a particular listening event does for a particular person. That is a measurement problem before it is a clinical one.

Here, music-listening intervention means receptive listening delivered for health or well-being; music therapy refers to practice within a therapeutic relationship led by a qualified music therapist; and digital therapeutics refers to software-based interventions developed and evaluated as therapeutic products. These terms overlap in practice but are not interchangeable.

Many systems organize music by symptom label, intended mood, acoustic property, cultural category, expert judgment, or user preference. Such descriptions structure a repertoire and screen out unsuitable choices, but they do not specify how a person evaluates a track while listening. This Perspective argues that listener-level aesthetic appraisal should be treated as a distinct, directly measured component of personalized music listening rather than inferred from track properties or collapsed into preference, familiarity, engagement, or therapeutic outcome.

Terminologically, listener-level refers to the person-specific level at which appraisal is modeled and accumulated across sessions, whereas event-level or event-specific refers to the individual listening occasion at which appraisal is elicited. Event-specific reports are therefore the measurement units from which a listener-level appraisal profile is updated; the two terms are related but not synonymous.

The issue is not whether contemporary systems adapt; it is what they adapt to. Reviews of music-based applications for mental health indicate that the feedback routinely collected consists of liking or star ratings, skips, completion and listening duration, and periodic mood check-ins (Hides et al., 2019; Leschallier de Lisle and Oudin, 2024). Within the digital-therapeutic and algorithmic-recommendation literature considered here, we are not aware of an evaluated system that routinely collects an event-specific first-person response asking whether a track was experienced as meaningful, moving, or aesthetically satisfying and uses that response as an explicit input to subsequent selection.

This narrower claim does not deny the longstanding person-centered tradition of clinical music therapy. Individualized music protocols for dementia, including Gerdner’s evidence-based protocol, draw on biographical knowledge, personal preferences, and observed responses to guide music selection in care settings (Gerdner, 2012). Nordoff–Robbins practice similarly places the therapist’s musical attunement, moment-to-moment observation, and responsive musical interaction within the therapeutic relationship at the center of clinical work (Nordoff and Robbins, 2007). These approaches already attend closely to person-specific response, but their observations are therapist- or caregiver-mediated and clinically situated. The present proposal addresses a complementary measurement and decision problem: whether the listener’s own event-specific appraisal can be elicited explicitly, distinguished from observer inference, and incorporated as a repeatable input into a selection loop. Direct self-report therefore complements, rather than replaces, therapist observation. Observer-mediated responses remain valuable for care, safety, and tolerability, but they are not interchangeable with first-person aesthetic appraisal. Roadmaps for music technology in health care have called for precisely this kind of clarity about what is measured and modeled (Agres et al., 2021).

We define aesthetic appraisal as the listener’s event-specific evaluation of music as meaningful, moving, resonant, coherent, or aesthetically satisfying. The proposal makes three contributions. First, it separates event-level appraisal from correlated but non-equivalent constructs. Second, it defines personalization around the construct meaning of feedback rather than around adaptivity alone. Third, it outlines an illustrative, testable implementation comprising a measurement model, parameterized priors, a Bayesian updating rule, a constrained selection policy, logging requirements, and stopping criteria, together with prospective evaluation against static matching.

Older adults are a theoretically informative test case because cohort repertoires, autobiographical associations, hearing and cognitive differences, and care-mediated listening increase uncertainty in fixed labels and behavioral traces. The present argument rests on a conceptual distinction between descriptions used to organize candidate music and evaluations reported by listeners during specific listening events. Repertoire-level descriptors may inform predictions of individual responses, but they do not themselves constitute measurements of those responses. Whether direct appraisal adds useful information beyond existing descriptors and feedback indicators is an empirical question addressed by the proposed validation framework, not a finding established by a previously constructed library.

## 2 Construct definition and boundaries

The repertoire-side component describes candidate tracks before a focal listening event: acoustic and structural features, cultural and historical descriptors, symptom or context tags, and expert judgments of quality, suitability, and accessibility. The term repertoire-side is operational—expert judgments remain human evaluations, not intrinsic properties of the musical object. These variables can guide retrieval, but a cultural label cannot determine whether a piece evokes recognition, memory, distance, ambivalence, or indifference in one listener. Enculturation shapes musical-emotion perception even where some cues transfer across traditions (Balkwill and Thompson, 1999).

Event-specific aesthetic appraisal is the listener’s evaluation of music as an expressive and meaningful object in a particular listening event. The broader candidate facet pool includes meaningfulness, being moved, emotional resonance, coherence, beauty, warmth, energy, and aesthetic satisfaction. These facets should initially be treated as related but non-identical dimensions rather than as a single reflective trait. Meaningfulness may depend on autobiographical association; being moved (Menninghaus et al., 2015) and emotional resonance concern the affective-aesthetic impact of the listening event; coherence concerns the perceived organization of the musical experience; and satisfaction represents an overall evaluative judgment. A total score should be used only if multilevel dimensional evidence supports such aggregation.

To make event- specific appraisal operationally testable, we propose a provisional three-item assessment for repeated use in personalized music-listening interventions. The items are: (1) “How meaningful was this music to you just now?”; (2) “How much did this music move or resonate with you just now?”; and (3) “How satisfying was the musical experience just now?” Each item is rated using five response options scored from 0 to 4: 0 = “not at all,” 1 = “slightly,” 2 = “moderately,” 3 = “quite a bit,” and 4 = “very much.”

The wording “just now” anchors each response to the immediately preceding listening event. This temporal anchoring does not, by itself, establish discriminant validity from music preference or familiarity, which requires empirical evaluation. The three items were selected to represent three complementary aspects of appraisal: personal significance, affective-aesthetic resonance, and overall evaluative satisfaction. They are intended as provisional indicators rather than as a finalized and fully validated scale. The three items are newly worded provisional indicators, not validated short forms or verbatim items from GEMS, AESTHEMOS, or BMRQ. Future development should include mapping their content to relevant constructs and items in existing instruments, expert review, and cognitive interviews with older adults to assess content coverage and comprehensibility; discriminant validity from liking and familiarity should then be tested empirically.

Appraisal is related to preference and reward but identical to neither. Preference concerns liking, choice, or desire to hear a track and is relatively stable; appraisal is event-specific and may diverge—a favorite track may fall flat on a given evening, and an unfamiliar piece may prove unexpectedly moving. Familiarity is prior exposure; autobiographical salience is the degree to which a track indexes personal history. Both raise the probability of strong appraisal but neither guarantees its valence: a track tied to loss may be highly salient and aversive. Engagement refers to attention and involvement; exposure refers to how much music was delivered; adherence describes continued protocol use. All are commonly logged, and all are contaminated in real settings: completion may reflect inattention, duration may reflect who controlled the device, and adherence is influenced by burden, habit, and social context. Therapeutic outcomes are distal changes in symptoms, function, or quality of life; appraisal is a proximal experiential variable proposed as a candidate mechanism, not an assumption. Music-induced emotion overlaps with appraisal but is not identical: a listener may recognize sadness, feel moved, and evaluate the experience as valuable, all at once. Appraisal is the evaluative layer, not the affective response itself.

Direct event-specific appraisal reports are the most construct-proximal indicators proposed here. In the present framework, these reports are obtained through the provisional three-item assessment administered after each listening event. Skips, replays, dwell time, movement, facial affect, vocalization, and physiological activation may indicate engagement, arousal, exposure, discomfort, or caregiver involvement, but none independently demonstrates aesthetic appraisal. Where direct report is unavailable, such signals may still support personalization—provided the system states what it measured and does not relabel observation as first-person evaluation.

Observer-mediated response assessment is therefore a complementary clinical tradition rather than an equivalent measurement of first-person appraisal. In a therapeutic setting, a qualified therapist may infer moment-to-moment response from changes in affect, bodily expression, vocalization, musical interaction, or engagement and may adapt the intervention accordingly. Such observation can be clinically meaningful and therapeutically consequential, but it is generated within a relationship and is shaped by the observer’s interpretation and clinical goal. It does not answer the same measurement question as the listener’s own event-specific evaluative report. Accordingly, supported self-report is treated as first-person only when the listener supplies the answer; therapist, caregiver, and behavioral observations are recorded and analyzed as separate care-response outcomes with their own reliability, validity, and, where relevant, inter-rater evaluation.

## 3 Older adults as an informative test case

Older adults are theoretically informative rather than merely a target population, because four sources of uncertainty converge to make fixed labels unreliable.

Musical memories cluster around adolescence and early adulthood (Krumhansl and Zupnick, 2013; Jakubowski et al., 2020), and musical engagement and preference themselves shift across the life course (Bonneville-Roussy et al., 2013). This licenses cohort-informed curation, but with an important limit: cohort membership predicts exposure, not evaluation. Within one birth cohort, the same formative-period repertoire may be experienced as nostalgic, indifferent, or aversive depending on individual biography. Age-related changes in auditory processing affect the perception of timbre, harmony, and fine spectral detail, so two listeners may not be receiving the same musical object even when given the same file. Cognitive load and working-memory differences further affect the perception of large-scale form, which bears directly on the coherence facet.

Care-mediated listening compounds the problem. A caregiver or institution may start, stop, or repeat music, so duration and replay may reflect workflow rather than listener response. Evidence should be labeled by source and administration mode: independent self-report, supported self-report, caregiver-proxy rating, or behavioral observation. Supported self-report remains first-person when assistance facilitates access or communication without supplying or interpreting the answer on the listener’s behalf. Its comparability with independent self-report requires evaluation. Proxy judgments and observations are indirect evidence and are not interchangeable with the listener’s own appraisal. Much of the strongest evidence comes from dementia care, where the Cochrane estimate for depressive symptoms (SMD −0.27) coexists with null effects on agitation (van der Steen et al., 2018). Neither the effect nor its absence should be generalized automatically to cognitively healthy older adults.

The appraisal-based pathway is intended for older adults able to understand the event-level questions and communicate their own responses, independently or with appropriate support. Eligibility depends on reporting ability at the relevant event, not on a dementia diagnosis alone. When interpretable self-report is unavailable, an observational care pathway may support care-oriented personalization, but direct aesthetic appraisal remains unmeasured. The dementia intervention evidence cited above motivates the care context; it does not validate the proposed appraisal measure in people with dementia.

We propose a testable hypothesis: cohort membership may leave substantial between-listener variation in event-level appraisal unexplained, even when listeners encounter the same tracks. A suitable study would collect repeated appraisals from listeners in multiple prespecified cohorts using a shared or sufficiently overlapping track set, and distinguish cohort differences, listener differences within cohorts, track effects, contextual effects, and listener-by-repertoire interactions in a multilevel analysis. Large within-cohort differences would caution against treating cohort labels as sufficient descriptions of individual appraisal, but would not by themselves establish the benefit of adaptive selection. That benefit requires out-of-sample comparison with well-specified cohort-based and static person-level models, accounting for assessment burden. This is a prospective hypothesis and study design, not a result reported here or inferred from an unpublished library study; it can be evaluated in any suitably documented repertoire with an appropriate sampling design.

## 4 Relation to existing constructs and instruments

Psychological theories of musical emotion already distinguish aesthetic judgment from mechanisms such as brainstem reflex, entrainment, conditioning, contagion, imagery, episodic memory, and expectancy (Juslin and Västfjäll, 2008; Juslin, 2013), and music-reward research shows that value emerges through listener–music interaction (Salimpoor et al., 2011, 2013). Validated instruments exist for music-evoked emotion (GEMS; Zentner et al., 2008), aesthetic emotion (AESTHEMOS; Schindler et al., 2017), and music-reward sensitivity (BMRQ; Mas-Herrero et al., 2013).

Why, then, a new construct? Because three properties are required jointly, and no existing instrument supplies all three. First, the appraisal is measured at the event level rather than as a trait: BMRQ characterizes persons; GEMS and AESTHEMOS characterize responses to a stimulus in a session, typically in laboratory or single-occasion designs. We do not necessarily propose an entirely new aesthetic phenomenon; rather, we specify an event-level measurement and decision construct for adaptive music-listening interventions. Second, closure of the loop: existing scales terminate in a score used for description or group comparison; here the score must be written back into the next selection decision, which converts a psychometric question into a policy question. Third, health-intervention constraints: appraisal must be optimized jointly with therapeutic goals, safety, sensory accessibility, autonomy, response burden, and repertoire diversity—constraints absent from aesthetics research. Existing scales are therefore the natural source of candidate items, not a substitute for the construct proposed here.

## 5 A two-component closed loop

The repertoire-side component defines a quality-controlled candidate space through musical descriptors, cultural and contextual tags, expert curation, accessibility requirements, and intervention constraints. The listener-side component follows an appraisal-based pathway when interpretable first-person responses are available and an observational care pathway otherwise. In the appraisal-based pathway, direct appraisal responses update an appraisal profile, while preference, familiarity, autobiographical salience, engagement, exposure, and proximal response are measured separately. In the observational care pathway, separately labeled behavioral observations and caregiver reports inform a care-response profile for safety and tolerability, not an aesthetic-appraisal score. Both pathways inform subsequent selection within repertoire-side constraints while preserving uncertainty, safety, autonomy, and intervention goals. The observational pathway is a proposed care-oriented extension requiring separate validation, not a substitute for direct appraisal measurement. Proposed pathways from appraisal to engagement, meaning-making, affect regulation, exposure, adherence, or distal outcomes are hypotheses, not established mediation. Expert and listener judgments are complementary and non-substitutable: curation protects quality, suitability, and safety, while listener data estimate person-specific fit. The failure mode we are guarding against is not the absence of adaptation but the substitution of one side for the other—inferring person-specific fit from repertoire-level labels, or treating a listener’s accumulated behavioural traces as evidence that curation was unnecessary.

### 5.1 A minimally adequate measurement model

Listener-level aesthetic appraisal is provisionally represented by a family of related event-level dimensions. The listener-level appraisal profile is updated from repeated event-specific reports rather than treated as a single stable trait. Before administering the three core items, the assessor should briefly check whether the listener can understand the questions, relate them to the just-completed event, and communicate a response. This task-specific feasibility check does not establish psychometric reliability or constitute a cognitive diagnosis. When feasible, assessment should occur immediately after listening or within a short, prespecified interval. Neutral support may include reading items aloud, using large-print response options, or allowing pointing. Administration mode and assistance should be recorded; altered wording or response formats require separate evaluation before scores are pooled with the original format.

Unanswered or uninterpretable items should be recorded as missing, with reasons documented where known, rather than scored as zero. When interpretable self-report cannot be obtained, predefined observations of distress, apparent comfort, engagement, and refusal, together with caregiver reports, may be recorded for the observational care pathway. Any caregiver-proxy appraisal rating should be labeled as the caregiver’s inference. Neither proxy ratings nor observations should replace responses to the three self-report items.

The system should first record whether the listening event was completed, interrupted, rejected, or assisted by a caregiver. These behavioral and contextual variables should be stored separately from the listener’s direct appraisal responses.

To make the Bayesian component operational at a schematic level, we propose an illustrative hierarchical ordinal model, not a fitted or validated algorithm. Let *Y*_*itk*_ ∈ {0,1,2,3,4} denote listener *i*′s response to core item *k* = 1,2,3 at listening event *t*, when track *j*_*it*_ is presented. The three items are modeled separately, without assuming a validated total score. For each item, the cumulative-logit likelihood is Equation 1:


$$\text{Pr}\,\left(Y_{i\,t\,k}\leq r|\theta_{k}\right)={\text{logit}}^{-1}\,\left(\kappa_{k\,r}-\eta_{i\,t\,k}\right),r=0,1,2,3,$$

$$\eta_{i\,t\,k}=x_{i\,t}^{⊤}\beta_{k}+u_{i\,k}+v_{j_{i\,t}\,k}+q_{i,g\,\left(j_{i\,t}\right),k}.\;\left(1\right)$$

Here, *θ*_*k*_ denotes the parameters for item *k*, and *g*(*j*) denotes track *j*′s repertoire cluster, specified below. The vector *x*_*it*_ contains prespecified predictors available before selection, including track descriptors, listening context, and separately measured preference and familiarity. Continuous predictors are standardized using fixed calibration statistics. The terms *u*_*ik*_, *v*_*jk*_, and *q*_*igk*_ represent listener, track, and listener-by-repertoire-cluster effects, respectively. No intercept is included because location is represented by the ordered thresholds κ_*k*0_ < κ_*k*1_ < κ_*k*2_ < κ_*k*3_. Category probabilities are obtained by differencing adjacent cumulative probabilities, with boundary probabilities zero and one.

An illustrative prior specification is β_*kh*_∼*N*(0,1), $u_{i\,k}∼N\,\left(0,\tau_{u,k}^{2}\right)$, $v_{j\,k}∼N\,\left(0,\tau_{v,k}^{2}\right)$, and $q_{i\,g\,k}∼N\,\left(0,\tau_{q,k}^{2}\right)$, where *h* indexes predictors and each standard deviation has a half-normal *N*^+^(0,1) prior. The threshold vector has the joint distribution obtained by sorting four independent draws from *N*(0,2^2^) in increasing order. Parameter families have independent priors, and random effects are conditionally independent given their standard deviations. These priors are illustrative starting specifications requiring prior-predictive checks and sensitivity analyses, not validated defaults.

The mapping *g*(*j*) assigns each track to one of *G* mutually exclusive repertoire clusters independently of appraisal outcomes. One implementable option is k-means clustering of a prespecified, standardized set of acoustic and structural descriptors, fitted before adaptive data collection. The descriptor set, preprocessing and scaling statistics, number of clusters, initialization seed, fitted cluster centers, and track-to-cluster assignments must be fixed and reported for replication. Cultural and contextual tags may remain separate predictors, while safety and accessibility restrictions are applied as selection constraints rather than treated as evidence of appraisal.

Cluster assignments and model parameters are assumed stable within the prespecified adaptation window. Repeated responses are assumed conditionally independent given the predictors and random effects. Calibration should include multiple tracks per cluster and overlapping exposure across listeners to help distinguish track effects from listener-by-cluster effects. Sparse coverage and residual temporal dependence require diagnostic assessment; extensions for time-varying effects would need separate specification and evaluation.

The broader facets—including warmth, energy, coherence, beauty, and related descriptors—may be assessed during initial instrument development, baseline characterization, or periodic calibration. They need not all be administered after every listening event. This separation between core repeated items and supplementary facets is intended to preserve conceptual breadth while limiting respondent burden.

The proposed items require subsequent psychometric evaluation before they can be used as the primary basis for adaptive music selection. Relevant criteria include event- specific response reliability, within-person reliability, sensitivity to change across listening events, construct validity, discriminant validity from preference and familiarity, and incremental predictive validity for proximal listener responses. Feasibility should also be assessed in terms of comprehension, completion time, missing responses, response burden, and cognitive and sensory accessibility for older adults.

Listener-by-repertoire-cluster variance characterizes one source of appraisal heterogeneity, but it does not directly quantify the practical benefit of updating. In the ordinal model above, the ratio of listener-by-cluster variance to listener variance is defined on the latent logit scale, rather than the observed appraisal or clinical-outcome scale. The same ratio can imply different selection benefits depending on absolute effect sizes, response thresholds, differences between candidate tracks, calibration data, and the selection policy and constraints. A transferable numerical benchmark from other intervention settings has not been established for this application. We therefore do not use a fixed variance ratio as a universal rejection criterion. The value of adaptation should be assessed against a prespecified static comparator using future-event prediction and prospectively measured listener outcomes, with the minimum useful improvement and acceptable assessment burden defined before evaluation. Neither interaction variance nor posterior concentration alone establishes whether adaptive selection is worthwhile.

### 5.2 An operational specification

The appraisal-specific updating rules below apply only to events with interpretable first-person appraisal data. When these data are unavailable, no observed appraisal score is entered, and behavioral or proxy data are not used to impute it. The appraisal profile receives no new appraisal evidence from that event, and uncertainty should not be reduced as if appraisal had been observed. Separately recorded care-response indicators may inform conservative selection for safety and tolerability under caregiver or clinician oversight. Reasons for missing appraisal and changes in reporting ability should be logged because missingness may be informative. Independent and supported self-reports should initially be distinguished in analysis; pooling requires evidence of measurement comparability. Proxy and observational data should be analyzed as separate outcomes.

For each item *k*, let *D*_*t – 1,k*_ denote the interpretable appraisal responses available before the next update, together with their recorded predictors and track assignments. Under the model specified in Section 5.1, the sequential posterior update is Equation 2:


$$\begin{array}{cccc} & p\left(\theta_{k}|D_{t,k}\right)\propto p\left(y_{i\,t\,k}^{,}{|}^{,}\theta_{k}j_{i\,t}x_{i\,t}\right)p\left(\theta_{k}|D_{t-1,k}\right). & & \text{(2)} \end{array}$$

At the first update, the second factor is the specified prior. Missing items contribute no appraisal likelihood factor, and proxy or behavioral observations do not replace them. Analysis of the observed responses assumes that missingness is ignorable conditional on recorded information; potentially informative missingness requires sensitivity analysis. Partial pooling allows population information to inform a new listener’s initial profile while retaining uncertainty about individual effects. Posterior uncertainty need not decrease after every observation.

For a minimal selection rule, prespecify one target item k* according to the listening objective rather than assuming that the three items form a validated total score. For each candidate track, define the normalized posterior-predictive expected appraisal as $U_{i\,j\,t}=E\left(Y_{i,t+1,k^{*}}^{,}{|}^{,}D_{t}jx_{i,t+1}\right)/4$, where the candidate-specific predictors are known before selection. Let *E*_*it*_ be the nonempty set of tracks remaining after safety, accessibility, intervention-goal, and listener-veto restrictions. Select track *j* with probability:


$$\begin{array}{cccc} & \pi_{i\,t}\,\left(j\right)=\left(1-\epsilon \right)\,\frac{\exp\left(U_{i\,j\,t}/T\right)}{\sum_{\ell \in E_{i\,t}}\exp\left(U_{i\,\ell \,t}/T\right)}+\frac{\epsilon }{|E_{i\,t}|},j\in E_{i\,t}. & & \text{(3)} \end{array}$$

Here, 0 < ϵ < 1 controls uniform exploration and *T* > 0 controls how strongly selection favors higher predicted appraisal. An illustrative pilot setting is ϵ = 0.10 and *T* = 0.20, subject to simulation and feasibility evaluation. The exploration weight ϵ is a policy parameter, not a variance ratio or a rejection criterion; neither ϵ nor *T* is an empirically validated default. Every eligible track has a probability floor of ϵ/∣*E*_*it*_∣; ineligible tracks have probability zero. No selection occurs if the eligible set is empty. This rule targets predicted appraisal, not demonstrated therapeutic benefit, and its exploration component does not itself constitute uncertainty-optimal selection.

At each selection, record the eligible set, posterior-predictive utilities, policy parameters, model version, assigned selection probabilities, presented track, and any caregiver or clinician override. Overrides must be distinguished from policy-generated selections. These records support evaluation of adaptive exposure but do not alone establish causal identification; causal analyses additionally require an appropriate design and assumptions concerning confounding, positivity, and missing outcomes.

Computational convergence and participant-level stopping are distinct. An illustrative MCMC implementation uses four chains and checks rank-normalized $\hat{R}<1.01$ and bulk and tail effective sample sizes of at least 400 for monitored parameters and decision-relevant predictions; Hamiltonian Monte Carlo implementations should also show no divergent transitions. Failed diagnostics suspend model-based updating of the selection policy pending review. Posterior-predictive checks remain necessary because sampler convergence does not establish model adequacy.

Participant-level calibration should have a prespecified event cap and an early-stopping rule. As an illustrative feasibility setting, use at most 20 scored events per listener, with early stopping permitted after at least two scored events in each prespecified eligible cluster if the 95% posterior credible interval for the expected target-item response has width no greater than 0.5 points on the 0–4 scale for every currently eligible track. The initial eligible repertoire and number of clusters must permit this coverage within the cap. These numerical settings require simulation and feasibility evaluation rather than being treated as validated clinical thresholds. Distress, refusal, fatigue, or withdrawal overrides any precision target. Reaching the cap without adequate precision is recorded as insufficient information, not convergence; stopping calibration neither demonstrates therapeutic benefit nor necessarily ends otherwise appropriate listening.

## 6 Three testable questions, and the conditions for rejection

Q1 and the appraisal-profile component of Q2 should initially be tested in participants able to provide interpretable self-report. Results should be examined by cognitive status and reporting mode rather than generalized automatically to all older adults. Outcomes from the observational care pathway cannot establish the incremental validity of first-person aesthetic appraisal. That pathway requires separate evaluation of observational reliability, proxy-related bias, safety, and care outcomes.

**Q1—Incremental validity.** (Does event- specific appraisal predict proximal response beyond preference, familiarity, salience, engagement, and exposure? Reject if appraisal adds no meaningful predictive increment over those variables in cross-validated multilevel models—that is, if its unique contribution is indistinguishable from zero once cheaper indicators are included. In that case, appraisal is redundant and the behavioral traces currently in use are adequate.

**Q2—Value of iteration.** Do iteratively updated probabilistic profiles improve future-appraisal prediction and listener outcomes over a prespecified static baseline at comparable assessment burden? Assess prediction through forward-in-time validation without access to future responses, and evaluate selection-policy benefit prospectively. A practically useful advantage is not supported if the upper uncertainty bound for improvement falls below the prespecified minimum useful difference; imprecise results that do not resolve this comparison remain inconclusive. Interaction variance and posterior concentration alone do not demonstrate adaptive benefit.

Before prospective evaluation, simulation should examine how appraisal heterogeneity translates into selection benefit under the model in Section 5.1 and the policy in Section 5.2. Prespecified scenarios should vary listener-by-cluster variance from zero upward and, for positive listener variance, include variance ratios below, at, and above 0.10. Absolute listener variance, response thresholds, track effects, calibration length, missingness, and exploration settings should also be varied. Here, 0.10 is only a sensitivity-analysis point, not an acceptance or rejection boundary. Adaptive and static policies should receive the same initial information and use the same eligibility constraints and assessment schedule; the static policy should retain its initial fitted profile, whereas the adaptive policy incorporates subsequent appraisal responses.

Using the prespecified target-item utility in Section 5.2, a simulated assignment can be classified as meaningfully suboptimal when its true expected utility falls more than a prespecified margin below that of the best eligible track at that event. Outputs should include the expected number of such assignments per session, the reduction achieved by adaptation relative to static matching, cumulative expected utility loss relative to the best eligible track, and assessment burden. The utility margin and session length should be prespecified, with sensitivity analyses across plausible margins and Monte Carlo uncertainty reported. This is a proposed simulation specification, not an analysis conducted for this Perspective; it characterizes performance under stated assumptions but cannot establish empirical base-rate expectations or clinical benefit.

**Q3—Trade-offs.** How should aesthetic fit be balanced against intervention goals, safety, autonomy, and burden? Reject the closed loop as specified if the measurement burden required for stable estimates reduces adherence or well-being more than improved matching benefits it—a net-effect question that must be evaluated empirically, not assumed away.

These are stated as rejection conditions deliberately. A framework that cannot fail is not a contribution.

To make the proposed updating and selection process concrete, consider a hypothetical 78-year-old listener, L1, who can provide interpretable event-specific self-reports after each listening session. For illustration, assume that the three provisional appraisal items are scored from 0 (“not at all”) to 4 (“very much”): meaningfulness, emotional or aesthetic resonance, and overall satisfaction. The hypothetical ratings across the three listening sessions are presented in Table 1. These values are used only to illustrate the updating logic; they do not represent validated psychometric results or empirical estimates.

**TABLE 1:** Hypothetical event-specific appraisal data for listener L1.

Listening session	Track	Meaningfulness	Emotional or aesthetic resonance	Overall satisfaction
1	Track A	3	2	3
2	Track B	4	4	4
3	Track C	1	1	2
Descriptive mean	—	2.7	2.3	3.0

The values are hypothetical and are provided solely to illustrate the updating logic. They do not represent validated measurement results, fitted posterior estimates, or empirical findings.

As shown in Table 1, L1’s event-specific ratings vary across the three sessions. A simple descriptive summary of these observations gives mean scores of 2.7 for meaningfulness, 2.3 for emotional or aesthetic resonance, and 3.0 for overall satisfaction. These means are not themselves the listener-level profile or a substitute for the ordinal model specified in Section 5.1. The system should retain the individual event records, the relevant predictors, the administration mode, and the uncertainty associated with the limited number of observations.

Suppose, for illustration, that Track B has relatively high autobiographical salience and emotional expressiveness, whereas Track C is more energetic but less familiar to L1. After the third session, the accumulated observations provide provisional evidence that L1 may respond more favorably to music that is meaningful and emotionally resonant than to music selected primarily for high energy. The posterior-predictive utility specified in Section 5.2 could consequently increase for eligible tracks with related attributes, while the exploration component would preserve some probability for alternative tracks. Selection would nevertheless remain subject to safety, accessibility, intervention-goal, and listener-veto constraints.

This example also clarifies the distinction between event-level and listener-level appraisal. The ratings obtained after Tracks A, B, and C are event-specific observations. Their accumulation and uncertainty-aware updating produce a provisional listener-level profile, which informs—but does not mechanically determine—the subsequent selection. If later observations provide contradictory evidence, the profile should be updated again rather than treated as a fixed trait.

## 7 Ethical and safety considerations

Direct appraisal measurement raises obligations that trace-based personalization conveniently avoids. Three matter most for older listeners. First, consent must be continuous rather than one-off: in dementia care, proxy consent authorizes participation, but ongoing assent—observable willingness at each session—governs each individual listening episode, and withdrawal must require no justification. Consent to participate and the ability to provide appraisal data are separate requirements: proxy consent does not make a proxy judgment a first-person report, and observable assent to listening does not establish the ability to complete the appraisal assessment. Second, autobiographically salient music is powerful precisely because it is not neutral; reminiscence-linked tracks can evoke grief or agitation as readily as pleasure. Adaptive systems must therefore treat distress as a first-class signal that halts selection rather than as noise to be averaged away, and caregivers must retain override authority. Third, event-level appraisal data are affective records of an identifiable person. Collection should be minimal. For routine repeated assessment, the proposed three-item set—meaningfulness, emotional or aesthetic resonance, and overall satisfaction—is preferred over administering the broader facet pool after every listening event. This low-burden choice necessarily provides less complete coverage of the broader appraisal domain; its psychometric adequacy therefore remains subject to the evaluation described in Section 5.1. The appraisal data should be retained only for as long as necessary for the adaptation window and should not be repurposed for unrelated profiling. Finally, hearing loss makes accessibility a safety matter, not a comfort one: presentation levels must be individually verified rather than defaulted. A loop that optimizes fit while eroding the listener’s authorship of their own listening has failed on its own terms.

## 8 Conclusion

This Perspective proposes an auditable way to incorporate listener-informed aesthetic appraisal into personalized music-listening interventions. Its practical implication is a clear sequence: define a quality-controlled candidate space; apply safety, accessibility, intervention-goal, and listener-veto constraints; collect a minimal event-specific appraisal when interpretable self-report is feasible; update the listener-level appraisal profile while retaining uncertainty; and select subsequent tracks within the eligible set. This sequence provides a basis for evaluating whether appraisal-informed adaptation improves future selection without compromising autonomy, safety, or feasibility.

Three implementation principles follow from the framework. Measurement should precede optimization, because a system cannot meaningfully update on an undefined signal. Uncertainty should remain explicit, because early observations in older adults may be sparse, context-dependent, and often care-mediated. Adaptive selection probabilities, eligible sets, model versions, and caregiver or clinician overrides should be logged to support evaluation of adaptive exposure. Such records do not, by themselves, establish causal identification, which requires an appropriate design and explicit assumptions, concerning confounding, positivity, and missing outcomes.

The proposed implementation is illustrative rather than fitted or validated. Immediate research steps are to evaluate the provisional appraisal items through content review, cognitive interviewing, and repeated-measures psychometric studies; assess prior and policy behavior in simulation; examine listener-by-repertoire variation using repeated event- specific appraisaldata; and prospectively compare adaptive selection with static matching under comparable initial information, eligibility constraints, and assessment burden. These studies should evaluate not only predictive improvement but also comprehension, missingness, accessibility, burden, safety, and the comparability of independent and supported self-report.

The framework is deliberately open to negative or inconclusive findings. It should be reconsidered if direct appraisal provides negligible incremental predictive value, if adaptive selection fails to achieve a prespecified practically useful improvement over static matching, or if the burden or risks of repeated assessment outweigh the benefits of improved matching. The observational care pathway should likewise be evaluated separately and should not be interpreted as evidence that first-person aesthetic appraisal has been measured when it has not. Inadequately precise findings should remain inconclusive rather than being treated as evidence of either benefit or failure.

The contribution of this Perspective is therefore not a claim that the proposed loop is already effective. Rather, it is a testable measurement and decision specification for determining whether event-specific appraisal provides practically useful information for updating listener-level profiles and whether such updating improves subsequent music selection in older-adult care and well-being contexts.

**FIGURE 1:**
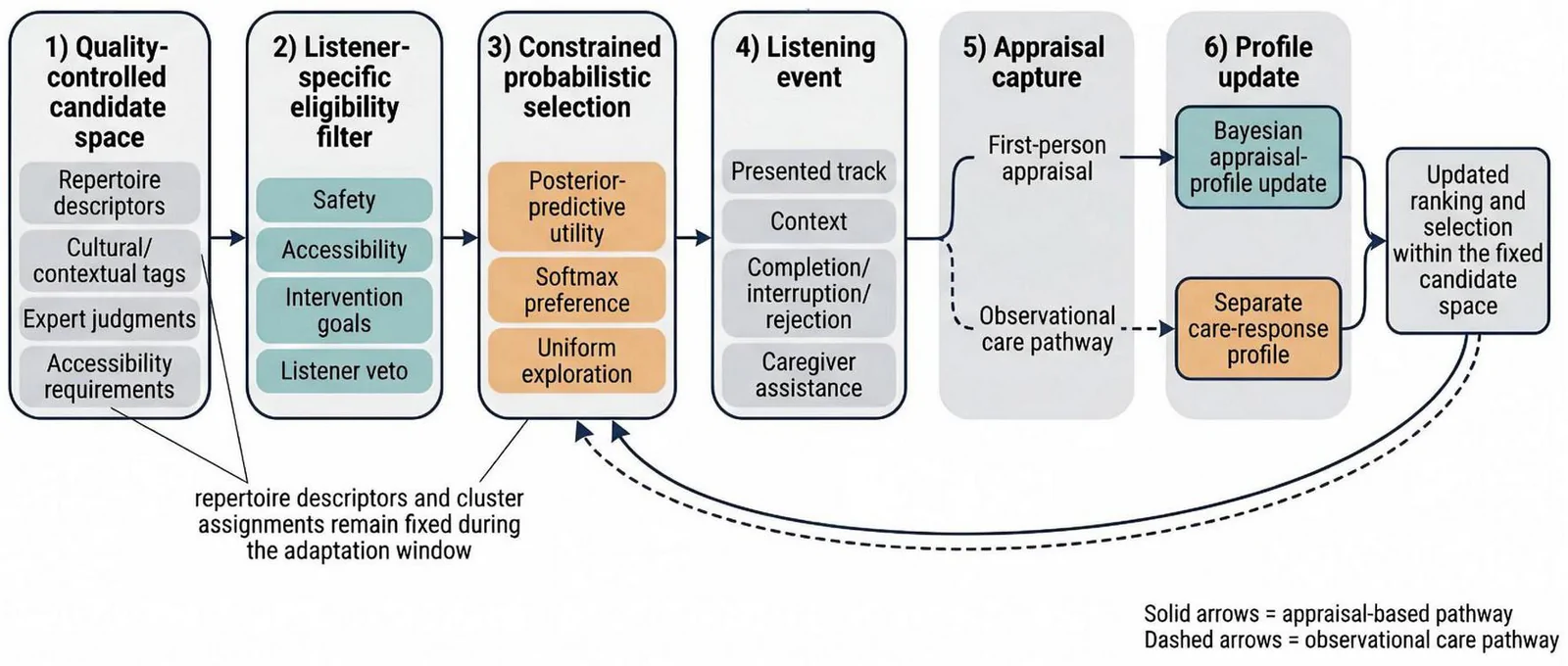
It summarizes the closed-loop sequence. Its feedback arrow denotes updated ranking and selection within the fixed repertoire-side candidate space; it does not imply that listener responses alter the underlying repertoire descriptors or cluster assignments during the adaptation window.

## Data Availability

The original contributions presented in the study are included in the article/Supplementary material, further inquiries can be directed to the corresponding author.
